# THE COMBINED EFFECTS OF LONELINESS AND SOCIAL ISOLATION ON MENTAL HEALTH IN A NATIONAL SAMPLE OF OLDER ADULTS

**DOI:** 10.1093/geroni/igac059.2619

**Published:** 2022-12-20

**Authors:** Melissa Krook, Corey Mackenzie, Verena Menec, Jessica Cameron

**Affiliations:** University of Manitoba, Winnipeg, Manitoba, Canada; University of Manitoba, Winnipeg, Manitoba, Canada; University of Manitoba, Winnipeg, Manitoba, Canada; University of Manitoba, Winnipeg, Manitoba, Canada

## Abstract

Social connections are important to maintain health across adulthood. Loneliness and social isolation are global issues and are linked to negative mental health outcomes worldwide, especially among older adults. Past research focuses primarily on loneliness and isolation separately, though many older people experience them simultaneously. Also, there is a paucity of research examining mechanisms through which combinations of loneliness/isolation result in poor mental health. My first objective examined how combined loneliness/isolation affect psychological distress among a group of older adults, and how grouping this sample into four groups of loneliness (yes/no) and isolation (yes/no) may help identify which group(s) are at the greatest risk for distress. My second objective explored perceived social support and relationship satisfaction as mediators of the effects of combined loneliness/solation on distress. I addressed these objectives with a cross-sectional national sample of 2,745 Canadian older adults, aged 55 to 101 years, who completed self-report measures of loneliness, social isolation, social support, relationship satisfaction, perceived physical health, and psychological distress. Those experiencing greater combined loneliness/isolation also experienced higher levels of distress. For lonely older adults, experiencing isolation simultaneously predicted clinically significant distress, but this was not true for those who were not lonely. Participants who were both lonely/isolated had the poorest mental health because they were less satisfied with their relationships, but not because they had less perceived social support. The present study has the potential to expand what we know about pathways through which combinations of loneliness/isolation may lead to poor mental health in older adults.

